# The Impact of Primary Care: A Focused Review

**DOI:** 10.6064/2012/432892

**Published:** 2012-12-31

**Authors:** Leiyu Shi

**Affiliations:** Johns Hopkins Bloomberg School of Public Health, 624 North Broadway, Baltimore, MD 21205, USA

## Abstract

Primary care serves as the cornerstone in a strong healthcare system. However, it has long been overlooked in the United States (USA), and an imbalance between specialty and primary care exists. The objective of this focused review paper is to identify research evidence on the value of primary care both in the USA and internationally, focusing on the importance of effective primary care services in delivering quality healthcare, improving health outcomes, and reducing disparities. Literature searches were performed in PubMed as well as “snowballing” based on the bibliographies of the retrieved articles. The areas reviewed included primary care definitions, primary care measurement, primary care practice, primary care and health, primary care and quality, primary care and cost, primary care and equity, primary care and health centers, and primary care and healthcare reform. In both developed and developing countries, primary care has been demonstrated to be associated with enhanced access to healthcare services, better health outcomes, and a decrease in hospitalization and use of emergency department visits. Primary care can also help counteract the negative impact of poor economic conditions on health.

## 1. Introduction

Primary care serves as the cornerstone for building a strong healthcare system that ensures positive health outcomes and health equity [1, 2]. In the past century, there has been a transition in healthcare from focusing on disease-oriented etiologies to examining the interacting influences of factors rooted in culture, race/ethnicity, policy, and environment. Such a transition called for person/family-focused and community-oriented primary care services to be provided in a continuous and coordinated manner in order to meet the health needs of the population. In 2001, the World Health Organization (WHO) proposed a global goal of achieving universal primary care in the six domains established by the 1978 Alma-Ata Declaration: first contact, longitudinality, comprehensiveness, coordination, person or family-centeredness, and community orientation. These six attributes, agreed upon internationally, have proved effective in identifying breadth of primary care services and monitoring primary care quality [3–6].

However, despite near consensus around the world that primary care is a critical component of any healthcare system, there is a considerable imbalance between primary and specialty care in the United States (USA) and many other parts of the world. For example, in the USA, in 2008, among 954,224 total doctors of medicine, 784,199 were actively practicing and 305,264 were practicing in primary care specialties (32% of the total and 39% of actively practicing physicians) [7]. The proportion of specialists was over 60% of all patient care physicians.

The major driving force behind the increasing number of medical specialists is the development of medical technology. The rapid advances in medical technology continuously expanded the diagnostic and therapeutic options at the disposal of physician specialists. The majority of patients, significantly freed from financial constraints thanks to third-party insurance payment, have turned to physicians who can provide them with the most up-to-date, sophisticated treatment. Hence, the rapid advance of medical technology contributes to the demand for specialty services and provides an impetus for further specialty development.

In addition, significantly higher insurance reimbursement for specialists relative to primary care physicians also contributes to the current imbalance. Under the resource-based relative value scale (RBRVS), implemented for US Medicare physician payment, primary care physicians continue to receive lower payments than specialists for comparable work because physician payments are based on historically determined, estimated practice costs as well as total work effort [8, 9]. Moreover, many insurance companies will pay for hospital-based complex diagnostic and invasive procedures using high technology, but not for routine preventive visits and consultations. Such practices not only encourage medical students' career choices in subspecialties and practicing physicians' provision of intensive specialty services, but also discourage the provision of important primary care services and deter patients from early care-seeking behavior.

Specialist physicians enjoy other benefits as well. Not only do specialists earn significantly higher incomes than primary care physicians, but also they are more likely to have predictable work hours and enjoy higher prestige both among their colleagues and from the public at large [10, 11]. Problems typically cited in recruiting primary care physicians include longer working hours during the day as well as on call, less financial reward for service, and less access to the highly technological approaches to diagnosis which is an important part of the medical center approach to patient care [12]. Among factors affecting medical students' career choice, society's perception of value, intellectual challenge, and lifestyle factors (e.g., hours worked) were ranked as very important along with financial reward [13–15]. The medical education environment, organized according to specialties and controlled largely by those who have achieved their leadership positions by demonstrating their ability in narrow scientific or clinical areas, emphasizes technology intensive procedures, and tertiary care settings also deter the choice by students of primary care specialties [16, 17].

Perhaps the most important reason for this imbalance is the lack of appreciation for the true value of primary care. Relative to disease-specific research, primary care-oriented studies have been relatively few. Their dissemination and recognition within the medical field are also problematic. Policymakers and the general public also have little knowledge of the efficacy of primary care, its impact on individual and population health, and its role in today's healthcare delivery. These realities have led to superfluous political commitments and the disengagement of related sectors [18, 19]. A WHO 2000 report announced that primary care has failed to serve as the foundation of care for all people [2].

The objective of this focused review paper is to present the research findings regarding the efficacy of primary care so that the value of primary care can be better appreciated. Specifically, it will demonstrate the importance of effective primary care services in delivering quality healthcare, improving health outcomes, and reducing disparities.

## 2. Methods

Literature searches were performed in PubMed using the following key search terms: primary care (also general practice, family medicine) and quality, performance, health outcome, and health equity. The search was limited to English language journals. The titles and abstracts of all papers identified by the electronic search were inspected. Papers that failed to satisfy the inclusion criteria were discarded. The resulting references were required to be related to primary care quality and outcome studies. Articles focusing on clinical procedures were excluded since the focus of this paper was on the general characteristics of primary care. Additional important articles were subsequently located by examining the bibliographies of the retrieved articles. The content areas to be reviewed include the following: primary care definitions, primary care measurement, primary care practice, primary care and health, primary care and quality, primary care and cost, primary care and equity, primary care and health centers, and primary care and healthcare reform.

### 2.1. Primary Care Definitions

The terms “primary care” and “primary healthcare” describe two different concepts. The former, primary care, refers to family medicine services typically provided by physicians to individual patients and is person-oriented, longitudinal care [20]. Primary healthcare, in contrast, is a broader concept intended to describe both individual-level care and population-focused activities that incorporate public health elements. In addition, primary healthcare may include broader societal policies such as universal access to healthcare, an emphasis on health equity, and collaboration within and beyond the medical sector [20].

Primary care plays a central role in a healthcare delivery system. Other essential levels of care include secondary and tertiary care, which encompass different roles within the health spectrum. Compared to primary care, secondary and tertiary care services are more complex and specialized, and the types of care are further distinguished according to duration, frequency, and level of intensity. Secondary care is usually short-term, involving sporadic consultation from a specialist to provide expert opinion and/or surgical or other advanced interventions that primary care physicians (PCPs) are not equipped to perform. Secondary care thus includes hospitalization, routine surgery, specialty consultation, and rehabilitation. Tertiary care is the most complex level of care, needed for conditions that are relatively uncommon. Typically, tertiary care is institution-based, highly specialized, and technology-driven. Much of tertiary care is rendered in large teaching hospitals, especially university-affiliated teaching hospitals. Examples include trauma care, burn treatment, neonatal intensive care, tissue transplants, and open heart surgery. In some instances, tertiary treatment may be extended, and the tertiary care physician may assume long-term responsibility for the bulk of the patient's care. It has been estimated that 75% to 85% of people in a general population require only primary care services in a given year; 10% to 12% require referrals to short-term secondary care services; 5% to 10% use tertiary care specialists [21].

Since its introduction in 1961, the term primary care has been defined in various ways, often using one or more of the following categories of classification [4, 22–24]. These categories include the following.

- The care provided by certain clinicians, the Clinton administration's Health Security Act, for example, specified primary care as family medicine, general internal medicine, general pediatrics, and obstetrics and gynecology. Some experts and groups have also included nurse practitioners and physician assistants.
- A set of activities whose functions act as the boundaries of primary care—such as curing or alleviating common illnesses and disabilities.
- A level of care or setting—an entry point to a system that also includes secondary care (by community hospitals) and tertiary care (by medical centers and teaching hospitals).
- A set of attributes, as in the 1978 IOM definition—care that is accessible, comprehensive, coordinated, continuous, and accountable—or as defined by Starfield [4]—care that is characterized by first contact, accessibility, longitudinality, and comprehensiveness.
- A strategy for organizing the healthcare system as a whole—such as community-oriented primary care, which gives priority and resources to community-based healthcare while placing less emphasis on hospital-based, technology-intensive, and acute-care medicine.

Definitions of primary care often focus on the type or level of services, such as prevention, diagnostic and therapeutic services, health education and counseling, and minor surgery. Although primary care specifically emphasizes these services, many specialists also provide the same spectrum of services. For example, the practice of most ophthalmologists has a large element of prevention, as well as diagnosis, treatment, followup, and minor surgery. Similarly, most cardiologists are engaged in health education and counseling. Hence, according to some experts, primary care should be more appropriately viewed as an approach to providing healthcare, rather than as a set of specific services [21].

The World Health Organization (WHO) describes primary care as essential healthcare based on practical, scientifically sound, and socially acceptable methods and technology made universally accessible to individuals and families in the community by means acceptable to them and at a cost that the community and the country can afford to maintain at every stage of their development in a spirit of self-reliance and self-determination. It forms an integral part of both the country's health system (of which it is the central function) and a main focus of the overall social and economic development of the community. It is the first level of contact for individuals, the family, and the community with the national health system, bringing healthcare as close as possible to where people live and work, and constitutes the first element of a continuing healthcare process [25].

Others define primary care as the health services rendered by providers acting as the principal point of consultation for patients within a healthcare system [26, 27]. This provider could be a primary care physician, such as a general practitioner or family physician, or (depending on the locality, health system organization, and the patient's discretion) a pharmacist, a physician assistant, a nurse practitioner, a nurse (as is common in the United Kingdom), a clinical officer (such as in parts of Africa), or an Ayurvedic or other traditional medicine professionals (such as in parts of Asia). Depending on the nature of the health condition, patients may then be referred for secondary or tertiary care. 

Perhaps the most comprehensive definition of primary care was given by Starfield in her landmark book *Primary care: balancing health needs, services and technology *[4]; Starfield defined primary care as the provision of integrated, accessible healthcare services by clinicians who are accountable for addressing a large majority of personal healthcare needs, developing a sustained partnership with patients, and practicing in the context of family and community. She summarized the following characteristics of primary care (pp. 19–34).

- Integrated care is intended to encompass the provision of comprehensive, coordinated, and continuous services that provide a seamless process of care. Integration combines information about events occurring in disparate settings and levels of care as well as over time, preferably throughout the life span.
- Comprehensive care addresses any health problem at any given stage of a patient's life cycle.
- Coordinated care ensures the provision of a combination of health services and information to meet a patient's needs. It also refers to the connection between, or the rational ordering of, those services, including the resources of the community.
- Continuous care is a characteristic that refers to care over time by a single individual or team of healthcare professionals (“clinician continuity”) as well as to effective and timely maintenance and communication of health information (events, risks, advice, and patient preferences) (“record continuity”).
- Accessible care refers to the ease with which a patient can initiate an interaction for any health problem with a clinician (e.g., by phone or at a treatment location) and includes efforts to eliminate barriers such as those posed by geography, administrative hurdles, financing, culture, and language.
- Healthcare services refer to an array of services that are performed by healthcare professionals or under their direction, for the purpose of promoting, maintaining, or restoring health. The term refers to all settings of care (such as hospitals, nursing homes, physicians' offices, intermediate care facilities, schools, and homes).
- A clinician is an individual who uses a recognized scientific knowledge base and has the authority to direct the delivery of personal health services to patients.
- Accountability is applied to primary care clinicians and the systems in which they operate. These clinicians and systems are responsible to their patients and communities for addressing a large majority of personal health needs through a sustained partnership with a patient in the context of a family and community and for (1) quality of care, (2) patient satisfaction, (3) efficient use of resources, and (4) ethical behavior.
- A majority of personal healthcare needs refer to the essential characteristic of primary care clinicians: that they receive all problems that patients bring—unrestricted by problem or organ system—and have the appropriate training to manage a large majority of those problems, involving other practitioners for further evaluation or treatment when appropriate. Personal healthcare needs include physical, mental, emotional, and social concerns that involve the functioning of an individual.
- Sustained partnership refers to the relationship established between the patient and clinician with the mutual expectation of continuation over time. It is predicated on the development of mutual trust, respect, and responsibility.
- A patient is an individual who interacts with a clinician either because of real or perceived illness or for health promotion and disease prevention.
- Context of family and community refers to an understanding of the patient's living conditions, family dynamics, and cultural background. Community refers to the population served, whether they are patients or not. It can refer to a geopolitical boundary (a city, county, or state), members of a health plan, or neighbors who share values, experiences, language, religion, culture, or ethnic heritage.

### 2.2. Primary Care Measurement

Measurement enables assessment of the performance of a healthcare delivery system and individual providers. Additionally, measurement facilitates efforts to improve accountability, quality, appropriate use of resources, and patient outcomes and to lower the risk of adverse events [28]. Measurement is also increasingly tied to healthcare financing through pay-for-performance programs. As the USA attempts to emphasize primary care functions through aspects of the Patient Protection and Affordable Care Act [29], measurement of primary care will take on even greater importance. Shi notes that assessments of the quality of primary care patients receive should consider the four dimensions of primary care: the first contact experience, longitudinality, coordination, and comprehensiveness [30].

Researchers can use various types of indicators depending on the goal of measurement [28]. Indicators can provide some sense of the structure, process, or outcome of care, can be used to measure activity, performance, and quality, and can help determine whether the care is being provided according to guidelines specified by an expert body or consensus [28]. 

The Primary Care Assessment Tool (PCAT) is a collection of questionnaires, developed by Johns Hopkins Primary Care Policy Center under the leadership of the late Dr. Barbara Starfield, that assess whether a healthcare provider or system is achieving the four core functions of primary care (first contact, longitudinality, comprehensiveness, and coordination) and three supplementary aspects of primary care (family centeredness, community orientation, and cultural competence). The first PCAT-adult questionnaire was developed and validated in the USA [31, 32] but its validity and reliability have been demonstrated in other countries, such as in Brazil [33] and Spain [34]. Several forms of the PCAT exist, varying in length and target population. For example, while the Primary Care Assessment Tool-Adult Edition's (PCAT-AE) original form includes 74 items assessing adult patient experiences with primary care [31, 32] a short 10-item version, the PCAT10-AE has also been used and integrated into a national population health survey [34]. A PCAT assessing the primary care experiences of children has been developed as well [33, 35]. In addition to these questionnaires targeting patients, versions of the PCAT have been developed that also survey providers and administrators of facilities, providing another perspective on the provision of primary care [36].

In addition to the PCAT collection of survey instruments, researchers have used other surveys to measure aspects of primary care provision from the patient and provider perspective in the USA and in international settings. These include the Health Tracking Physician Survey [37], the International Health Policy Survey [38], and the Ambulatory Care Experiences Survey [39]. Other studies have used claims data [40, 41] and medical record review [40, 42–44] to assess the quality, performance, and cost-effectiveness of primary care in various settings.

Medical experts have defined standards of practice for assessment of providers or facilities in terms of whether they are practicing according to recommended guidelines [38, 44]. For example, a survey fielded in five countries determined that the USA performed well in delivering preventive care according to clinical guidelines [38], hypothesizing that this result might be due to third party insurers' increasing emphasis on quality measurement using tools such as the National Committee for Quality Assurance's (NCQA) Healthcare Effectiveness Data and Information Set (HEDIS). In addition to HEDIS, other indicators, such as the Diabetes Quality Improvement Project [40], have been developed to support measurement of the quality of care provided in a primary care setting for a particular condition. Many measures of performance and quality in the healthcare setting are disease-specific. Given primary care's emphasis on patient-centered and comprehensive care, these disease-specific measures may not be most useful for the primary care context. Other measurement efforts attempt to move beyond condition-specific indicators. Hospitalization for ambulatory care sensitive conditions (ACSC), defined as “diagnoses for which timely and effective outpatient care can help to reduce the risk of hospitalization” [45], has been proposed as a way to assess access to care and as an outcome measure of the effectiveness of prior primary care intervention [41]. However, research has shown that ACSC-related hospitalizations may occur too infrequently and be too difficult to link with previous receipt of primary care to serve as a viable outcome measure [41]. On the other hand, increased access to healthcare services is accomplished through expanded insurance coverage, thus also enabling greater financial access to hospital resources. Therefore, studies using preventable hospitalizations as outcome measures to examine the impacts of primary care access should consider how that improved access is being facilitated [46]. Another survey attempting to identify good indicators asked physicians about the types of patient outcomes that they value as good indicators of primary care providers' performance; respondents identified nineteen indicators related to patients' physical functioning, physical pain, physical symptoms besides pain, clinical indicators, emotional distress, health behaviors, and general quality of life.

Other literature examines the measurement of primary care with respect to unique populations, particular models of care, or atypical settings [39, 47, 48]. One challenge is measuring care provided to complex patients (patients with multiple chronic conditions), given that disease-specific measures are ill-suited for this population [48]. Therefore, indicators of the continuity and coordination aspects of primary care provision are particularly important for assessing the quality of care this complex population experiences [48]. Given the increasing emphasis on patient-centered medical homes (PCMH), measuring the impact of multidisciplinary teams (in contrast to individual providers) may better elucidate the patient experience of care in PCMH settings [39]. However, NCQA standards to assess medical homes may not be appropriate for all practice settings; for example, the military health system confronts different challenges when establishing medical homes related to deployment and the frequent movement of patients and providers [47].

Finally, the facilitators and barriers to implementing quality measurement in primary care were systematically reviewed in a study on primary care in Canada [49]. Content analysis of the 57 English-language articles published between 1996 and 2005 identified seven common categories of facilitators and barriers for implementing innovations, guidelines, and quality indicators. The authors found that successful implementation of quality measures can occur but that success depends on the interaction of multiple factors, including measurement characteristics, promotional messages, implementation strategies, resources, the intended adopters, and the intraorganizational and interorganizational contexts. Research has also found that the nature of the relationship between the patient and PCP impacts patients' perception of the quality of care they are receiving [50] and correlates positively with measures of primary care provider performance [51]. However, while the quality of care patients receive may be heavily impacted by the strength of connection patients feel with their providers, research has found that patients generally do not feel well connected to their PCPs [51].

In summary, primary care measurement includes tools that assess many aspects of care: the extent to which a primary care setting fulfills the major components of primary care; the performance of the provider or facility; the quality of care patients receive; how facets of care delivery, such as various models of care, team approaches, and different settings, impact care. Tools to collect data include primary data collection from surveys and secondary analysis using claims data and medical chart abstraction. Given the nature of primary care practice, indicators that are patient-centered rather than disease-specific are likely going to be increasingly important in enabling a more accurate assessment of the care patients receive.

### 2.3. Primary Care Practice

Many countries place great emphasis on primary care and have developed strong primary care infrastructures [52–54]. Examples include Britain's National Health Service (NHS), which established Primary Care Trusts (PCT) that integrate primary and hospital-based care and comprise the bulk of the NHS budget [52, 55]. Canada has a more balanced primary care-specialist physician ratio than the USA with only 10% more specialists than primary care physicians, in contrast to over 50% more in the USA [56]. Developing countries, like Brazil and Thailand, have also implemented national-level strategies to increase access to primary care services [57, 58].

An increasingly popular model for orienting the healthcare system to primary care is the gatekeeper model, which requires patients to select a primary care physician (PCP) and then obtain referrals through that PCP to specialists [59]. However, gatekeeper models may meet resistance from medical professionals and consumers in some countries [60]. Therefore, efforts to promote gate keeping in a healthcare system should consider gradual, incentive-driven approaches [60]. 

In conjunction with acting, in some systems, as gatekeepers to more specialized services, PCPs also may serve as patients' point of first contact with the healthcare system. Many countries have expanded access to primary care by establishing call centers, flexible hours, and clinics. Spain, for example, has sought to make care accessible both in financial and geographic terms, by enacting universal insurance coverage and striving to make healthcare facilities available within fifteen minutes to every person in need [61].

Continuity of care is also promoted through structures such as medical homes or well-developed health information technology (health IT) systems [59]. In Spain, for example, nearly every resident has an identification card that enables providers to access their medical history and relevant information at an appointment or emergency [61]. These countries have also sought to raise the status of primary care by establishing the discipline as a specialty within medicine and instituting reforms to payment systems [59].

Team-based models of providing primary care and the connections of these models with quality are becoming increasingly important as insurers use pay-for-performance incentives in payment schemes [62]. In order to support high-functioning teams, the associations between team-level job satisfaction and performance should be explored, a relationship which may be affected by the status and support enjoyed by the PCPs in a setting [62]. Research also suggests that the functioning level of primary care teams may affect patient outcomes, with those patients cared for by high functioning primary care teams experiencing better health outcomes [63]. Team-based approaches to primary care may also facilitate integration of mental health and primary care. As an example, the USA-based Intermountain Healthcare's mental health integration system includes PCPs, psychiatrists, nurses, family members, and other parties to integrate mental health services into the usual practice of primary care [64]. 

Scope of practice, the extent of health insurance coverage in a region, ease of coordination with other sectors, and myriad other factors impact the way in which primary care is practiced in a country, region, or individual practice. Countries that have enacted reforms that build on their existing primary care infrastructures can serve as case studies for the USA, where the ongoing implementation of the Patient Protection and Affordable Care Act (ACA) seeks to enhance the role of primary care in the US healthcare system. 

Currently, in contrast to some of its industrialized peers, the US healthcare system is much more heavily skewed toward specialty care [56, 58]. Although 51.3% of office visits were to primary care physicians in 2008, only about one-third of practicing physicians specialize in primary care [65]. A combination of primary care physicians, nurse practitioners (NPs), and physician assistants (PAs) comprise the estimated 400,000 primary care providers in the USA, with physicians contributing the largest portion (74%) [66]. Scopes of practice for NPs and PAs have broadened in many states in recent years, enabling these providers to take on more responsibilities in the provision of care. However, the distribution of primary care providers in the USA is uneven, with 5,902 communities designated as primary care health professional shortage areas [67].

Changes to the Medicare fee schedule (which had previously favored specialists in reimbursement rates) [68], support for Title VII health professions training programs [69, 70], and the recent ACA are some examples of policies that have attempted to strengthen the role of primary care within the US healthcare system. Some experts have suggested that the ACA and the aging population will place an increased burden on the primary care workforce in the USA, contributing to a severe workforce shortage in the future [71]. Although about one-third of practicing physicians work in primary care, less than a fourth of current medical school graduates are pursuing careers in primary care fields, and many primary care physicians are projected to retire in coming years, raising additional concerns that the future US primary care workforce will be unable to respond to the growing demand for primary care [72]. A factor contributing to the small percentage of graduating medical students that pursue residencies in primary care is the significantly lower salaries in these fields, a trend that has continued despite some efforts to reduce this disparity [71, 73]. 

Similarly, in order to incentivize providers to accept patients newly eligible for Medicaid under the reforms, the ACA temporarily raises reimbursement for PCPs serving Medicaid patients to the same level as Medicare reimbursements [74]. However, a study found that those states that have a low supply of PCPs serving Medicaid enrollees already have higher reimbursement levels [74]. Therefore, this increase may have little effect in increasing the supply of PCPs available to care for disadvantaged groups, such as the Medicaid population.

In order to address these fears, more research is needed on the capabilities and capacities of the current PCP workforce, as well as projections about how it will change over time. Indeed, a 2011 Robert Wood Johnson Foundation (RWJF) report observes that workforce projections are complicated [66]. The report cautions that although the workforce is likely to be strained by the country's changing demographics and increasing demand under the ACA, other clinicians, such as NPs and PAs, in addition to new team-based models of care, may change primary care workforce needs in unanticipated ways [66]. Nevertheless, the irregular distribution of providers in the USA remains a significant issue that is likely to continue inhibiting access to primary care services among particular segments of the population and in certain geographic regions [66].

Next steps and future directions have been identified to strengthen the primary care infrastructure abroad and in the USA. To start, there has been increasing interest in exploring how primary care and public health might better coordinate in order to support population health improvement efforts [75, 76]. A review of literature on the coordination of primary care with public health suggests that combinatory efforts can lead to improvements in the management of chronic diseases, control of communicable diseases, and in maternal and child health [76]. In addition, there is need for additional clarification on the unique roles of primary care and public health and the ways in which these sectors can work together [77]. 

In the USA in particular, new models of delivering care through patient-centered medical homes (PCMHs) and accountable care organizations (ACOs) require team-based approaches to care with a heavy emphasis on primary care. As previously discussed, some experts suggest that the shift to these models for delivering care will require an increased supply of primary care providers [58] whereas others note that little is definitively known about how these models of care will impact provider productivity [66]. This renewed interest in improving primary care capacity has led to some recommended initiatives for enhancing the stature of primary care in the USA, including increasing Title VII funding to better support the education of primary care providers that agree to practice in underserved communities [69, 70]; addressing salary disparities between PCPs and specialists by changing Medicare's resource-based relative value scale to give more equal reimbursement, which also influences private insurance reimbursement rates [78]; exploring the role that other primary care providers, such as NPs and PAs, can play in reducing burdens on primary care physicians [66]. Additional research is obviously needed; topics that should be examined include the methods and tools for conducting research on primary care, clinical issues of relevance to the practice of primary care, primary care service delivery, health systems (including the social and political factors affecting primary care provision), and how to improve the education and training of primary care providers [54].

### 2.4. Primary Care and Health

Logically, primary care is seen as an important medical specialty and healthcare necessity because it is assumed to have a positive impact on health outcomes; the USA and most other countries believe that increasing the quality and quantity of primary care services will lead to better population health. A number of ecological studies have examined the relationship between primary care infrastructure and health outcomes internationally [79–83] as well as in the USA at various levels of geographic units [84, 85]. Studies conducted in industrialized countries, such as member nations of the Organization for Economic Cooperation and Development (OECD), do indicate that stronger primary care systems are generally associated with better population health outcomes including lower mortality rates, rates of premature death and hospitalizations for ambulatory care sensitive conditions, and higher infant birth weight, life expectancy, and satisfaction with the healthcare system [79, 80, 82, 86]. Studies in the USA have also indicated that greater primary care availability in a community is correlated with both better health outcomes [87] and a decrease in utilization of more expensive types of health services, such as hospitalizations and emergency department (ED) visits [88]. 

Experiences in the international context suggest that primary care-oriented healthcare delivery systems can produce better health outcomes [52–55] in addition to counteracting, to some extent, the negative impact of poor economic conditions on health [57]. Reforms of healthcare systems to emphasize primary care generally are associated with improved health outcomes, including evidence from several countries in Latin America and Asia [83]. However, given that these reforms typically included multiple components, attributing change in population health to any one aspect of the reform is difficult [83]. Increasing primary care availability in low- and middle-income countries also correlates with improved health; however, many of these studies are limited to child and infant health outcomes [81]. Additionally, much of the research in this setting consists of observational studies rather than more rigorous research designs, and studies may also use different definitions of what constitutes a “primary care system” or “program” [81].

In a review of US primary care and its relationship with health outcomes, Starfield et al. [89] note that there may be several mechanisms of primary care that explain this positive association with population health: (1) better access to health services; (2) improved quality of care; (3) emphasis on prevention; (4) the identification and early management of conditions; (5) the combined impact of many characteristics of solid primary care systems; (6) reduction in unnecessary specialist care [89, 90].

Primary care and health service use were also studied in the USA using an interactional analysis instrument to characterize patient-centered care in the primary care setting and examine its relationship with healthcare utilization [91]. A total of 509 adult patients at a university medical center were randomized into groups receiving care by family physicians or general internists. An adaptation of the Davis Observation Code was used to measure patient-centered practices; the main outcome measures of the study were the patients' use of medical services and accrued charges over one year. The results indicated that higher amounts of patient-centered care were related to a significantly decreased annual number of visits to specialty providers, less frequent hospitalizations, and fewer laboratory and diagnostic tests. Total medical charges for the year were also significantly reduced.

Another US study examined the relationship between physician-patient connectedness and measures of physician performance [51]. 155,590 patients who made one or more visits to a study practice from 2003 to 2005 in the Massachusetts General Hospital adult primary care network were identified, and a validated algorithm was used to connect patients to physicians or practices. Performance measures, including breast, cervical, and colorectal cancer screening in eligible patients, hemoglobin A1C measurement and control in patients with diabetes, and low-density lipoprotein cholesterol measurement and control in patients with diabetes and coronary artery disease, were used to examine clinical performance. The results indicated that physician-connected patients were significantly more likely than practice-connected patients to receive guideline-consistent care. Receipt of preventive care varied more by whether patients were more or less connected to a primary care physician than by race or ethnicity, which are often cited as major determinants of healthcare usage. 

The role of primary care in referral was studied in a multicountry project in Europe and Australia [92]. The study compared weight loss achieved through standard treatment in primary care versus weight loss achieved after referral by the primary care team to a commercial provider in the community. In this parallel group, nonblinded, randomized controlled trial, 772 overweight, and obese adults were recruited by primary care practices in Australia, Germany, and the UK to receive either 12 months of standard care, as defined by national treatment guidelines or 12 months of free membership in a commercial program; analysis was by intention to treat amongst the population who completed the 12-month assessment. The results showed that the participants referred to community-based commercial providers lost more than twice as much weight over the year as compared to those who received standard care. These results indicate that referral to a commercial weight loss program that provides regular weighing, advice about diet and physical activity, motivation, and group support can offer a clinically useful early intervention for weight management in overweight and obese people and can be delivered on a large scale as well. However, it also demonstrates that primary care physicians and teams have limits in the scope and quality of interventions they can provide; in this case, the primary care team provided better care through the referral to an outside company than through the team-managed care seen as standard.

The impact of primary care outreach was tested in a Canadian study [93] using a randomized, controlled trial design to evaluate the impact of a provider-initiated primary care outreach intervention as compared with usual care among older adults at risk of functional decline. The sample was comprised of 719 patients enrolled with 35 family physicians in five primary care networks in Hamilton, Ontario, Canada. The 12-month intervention, provided by experienced home care nurses from 2004 to 2006, consisted of a comprehensive initial assessment using the Resident Assessment Instrument for home care, collaborative care planning with patients, their families, and family physicians, health promotion activities, and referral to community health and social support services. The primary outcome measures were quality adjusted life years (QALYs), use and costs of health and social services, functional status, self-rated health, and mortality. The results for the mean difference in QALYs, overall cost of prescription drugs and services, and changes over 12 months in functional status and self-rated health were not statistically significant. Therefore, the results of this study do not support adoption of this particular preventive primary care intervention for this target population of high-risk older adults.

Another study conducted in Pittsburgh, Pennsylvania, examined the role of nurses in primary care [94]. This study evaluated findings from a trial treatment for behavioral problems in 163 clinically referred children from six primary care offices in Pittsburgh. Participants were randomized to be treated in either the on-site, nurse-administered intervention (PONI) in primary care or enhanced usual care (EUC) characterized by on-site diagnostic assessment and facilitated referral to a local mental health provider. The main outcomes were measured by standardized rating scales. The results showed that children randomized to the PONI intervention were significantly more likely to access their assigned treatment, received more direct treatment, adjunctive services, and a longer duration of treatment, and had greater levels of sibling participation than children assigned to receive EUC. These findings indicate that a psychosocial intervention for behavioral problems delivered by nurses in a primary care setting is feasible, improves access to mental health services, and has some clinical efficacy. Options for enhancing clinical outcomes may include multifaceted collaborative care interventions in the pediatric practice.

The impact of primary care on chronic disease management is the subject of much research. For example, a USA-based study examined the impact of a multifaceted intervention on cholesterol management in primary care practices [95]. The study used a practice-based trial to test the hypothesis that a multifaceted intervention consisting of guideline dissemination enhanced by a computerized decision support system (CDSS) would improve primary care physician adherence to the Third Adult Treatment Panel (ATP III) guidelines and improve the management of cholesterol levels. A total of 61 primary care families and internal medicine practices in North Carolina enrolled in the trial; 29 received the Third Adult Treatment Panel (ATP III) intervention and 32 received an alternate intervention (JNC-7). The ATP III providers received a personal digital assistant providing the Framingham risk scores and ATP III-recommended treatment. They examined 5,057 baseline and 3,821 follow-up medical records. The study reports the positive effect on screening of lipid levels and appropriate management of lipid level test results and concludes that a multifactorial intervention, including personal digital assistant-based decision support, may improve primary care physician adherence to the ATP III guidelines.

 In a US study that focused on diabetes disease management, researchers used a randomized, controlled trial to examine the relationships among patient characteristics, labor inputs, and improvement in glycosylated hemoglobin (A1C) level in a primary care-based diabetes disease management program (DDMP) [96]. A total of 217 patients with type 2 diabetes mellitus and poor glucose control were enrolled. The results showed that patients in the intervention group had significantly greater improvement in A1C level than the control group that received no additional disease management support. In multivariate analysis, no significant differences in A1C level improvement were observed when stratified by age, race/ethnicity, income, or insurance status, and no interaction effect was observed between any covariate and intervention status. Labor inputs were similar regardless of age, race/ethnicity, sex, or education and may reflect the nondiscriminatory nature of providing algorithm-based disease management care.

The role of primary care in preventive care has also been studied. In a study conducted in Spain on physical activity promotion by general practitioners, researchers sought to assess the effectiveness of a physical activity promotion program at 11 Spanish public primary care centers using 6-, 12-, and 24-month follow-up measurements [97]. They recruited 4,317 individuals (2,248 intervention and 2,069 control), and fifty-six general practitioners (GPs) were randomly assigned to intervention or standard care (control) groups. The primary outcome measure was the change in self-reported physical activity from baseline. The results indicated that general practitioners were effective at increasing the level of physical activity among their inactive patients during the initial six months of an intervention but the effect leveled off at 12 and 24 months. Only the subgroup of patients receiving repeat prescriptions of physical activity maintained gains over the long term.

Many people suffering from mental health issues also receive health services in a primary care setting [98]. In the USA, an evaluation of a Department of Veterans Affairs (VA) program establishing primary care clinics in underserved communities found that while these clinics did improve access to more general health services, without a specialty mental healthcare component, they did not effectively expand access to mental health services [99]. Research is mixed on whether psychotherapy and counseling in the primary care setting is cost-effective but it does suggest that patients may be more open to these strategies than to antidepressant prescriptions, and psychotherapy may be more effective in treating depression than counseling [98]. Research has found that while counseling in primary care is associated with short-term improvement and patient satisfaction, there is little evidence of its effectiveness, in comparison to usual care, in treating depression in the long run [100].

An overview of low- and middle-income countries found that 14 countries, including China, with comprehensive primary care (defined as >80% skilled birth attendance rates) experienced health gains compared with countries with more selective primary care approaches. These health improvements seemed to “depend on progression to comprehensive primary care with a reliable referral system linking to functioning facilities” [101, p. 958]. However, the study looked at countries as a whole and so could not account for within-country variation, and additionally, the study defined countries as having comprehensive primary care based only upon their skilled birth attendance rates, and other primary care attributes were not considered. 

In the USA, a growing body of research has focused on the impact of primary care supply, infrastructure, and models of care on health outcomes. A review of studies assessing the relationship between supply of PCPs and various outcomes, such as all-cause and disease-specific mortality, life expectancy, low birth weight, and self-rated health, found correlations at the state, county, and MSA levels [84]. Research also indicates that local supply of PCPs per capita, using radii around zip codes to define service areas, is associated positively with patient receipt of preventive health services and that this local primary care availability mediates, to some extent, the impact of socioeconomic factors on the receipt of preventive care [102]. In addition, according to one study, Medicaid-enrolled children who have access to high quality, family-centered primary care have both lower nonurgent and urgent hospitalization rates [103].

However, methodological challenges exist in conducting research linking PCP supply to population health. When doing these analyses, the ratio of primary care to specialist physicians may be a more appropriate measure than just physician supply [85]. For example, while a correlation exists between the PCP supply and health outcomes, there is no association between specialist supply and health outcomes [89, 90]. Therefore, using a measure of physician supply per capita, without consideration of the balance of primary care and specialist physicians, may skew findings.

In response to this policy-relevant research, next steps have included proposals to increase the supply of primary care physicians in the USA. Findings suggest that increasing the supply of PCPs by just one unit per 10,000 physicians might improve health outcomes by 0.66% to as much as 10.8%, depending on the outcome considered [84]. 

Further research is needed on which models of care produce the best health outcomes. While past research has indicated team-based care produces better outcomes in some settings, few studies have examined the use of teams in primary care practice [104].

Other issues will also continue to affect the relationship between primary care and health. Many experts believe that primary care will have to change practice models to improve patient outcomes and physician job satisfaction, as demonstrated in many of the previously mentioned researches. However, others have also argued that in order to revitalize primary care in the USA, major system-level change is needed, especially in the way that primary care physicians are compensated relative to specialists [105].

### 2.5. Primary Care and Quality

Ease of access, the clinical quality of the care, interpersonal aspects of care, continuity, and coordination all are important elements to consider when assessing primary care quality [106].

Research exploring access has found that factors can impede or facilitate access, such as the availability of after-hours care, the length of office wait time, travel time to an appointment, lack of a specific PCP at the site of primary care, and lower perceived flexibility in selecting a PCP [5, 107]. Level of access to primary care impacts other facets of quality as well. For example, improved access to primary care may also improve the continuity of care for patients with depression [108]. An evaluation of a PCP access program found that better access led to reduced emergency department use in the long term [109]. Family-centered primary care may also lower rates of nonurgent emergency department visits and hospitalizations for certain populations [110]. However, a relationship between other measures of quality of primary care and urgent hospitalizations has not been established [110, 111].

The structure of the primary care delivery system may also affect levels of access and quality. For example, one New Zealand study comparing nonprofit and for-profit primary care practices found that the nonprofit practices in the study offered increased access at a lower cost in addition to offering a more expansive array of services and instituting written policies related to quality management [112]. In the USA, patients may experience differing levels of quality of primary care depending on insurance type. In an analysis comparing quality of primary care across various managed care models (i.e., managed indemnity, point of service, staff-model HMO, etc.), managed indemnity models performed best on quality of primary care measures, followed by point of service and network-model HMO structures [113]. The motivations within the delivery system can affect patient care as well; in a study of the impact of pay-for-performance initiatives on the quality of primary care received by patients with chronic conditions, researchers found a positive quality association for patients with two of the three conditions studied [114]. 

US Medicare recipients have a choice between private managed care plans (through the Medicare Advantage program) and the traditional government-managed fee for service (FFS) plan. Research indicates that while most quality performance measures are superior in the traditional FFS program, enrollees in some private plans may have better financial access to care [115]. Research has also sought to identify the most appropriate site for delivering primary care to low-income populations in the USA; these studies have mixed findings, with vaccination rates higher in hospital outpatient settings but fewer delays in receiving care in physicians' offices [116]. Structural features of primary care practices, such as having an electronic health record (EHR) and holding regular meetings devoted to discussing quality issues, can also be associated with higher performance on Healthcare Effectiveness Data and Information Set (HEDIS) measures [117]. Much research has examined EHRs and other health IT systems in hospitals or acute care settings, but these tools can facilitate quality improvement efforts in primary care settings as well [118]. Researchers have also found that health IT infrastructure facilitates provision of care for chronic conditions in line with the Chronic Care Model [119]. However, there remain significant challenges in the implementation of IT due to lack of reimbursement by insurance plans and the learning curve experienced by practitioners with the new technology [120].

Another component of primary care quality is continuity, defined as person-focused care over time. The following factors may affect the extent to which continuity can be achieved: appointment wait time length, the insurance status of patient, and after-hours care availability [5]. A review of studies conducted in 6 countries regarding primary care quality suggests that better continuity may decrease hospitalizations and ED visits, lowering healthcare costs [121]. 

In order to facilitate quality improvement in the primary care context, information is needed on appropriate benchmarks that can be used to evaluate performance in primary care-specific settings. Wessell et al. [122] identify Achievable Benchmarks of Care (ABCs) for 54 quality indicators based on data collected through the Practice Partner Research Network (PPRNet) demonstration. Twenty-five to 99% of the PCPs participating in the PPRNet demonstration met the ABCs [122]. A New Zealand effort identified 28 evidence-based, population-focused indicators that may be used to assess quality of primary care in five categories: smoking cessation, prescribing practices, chronic disease management, preventive health, and quality of data [123]. Furthermore, many of these indicators are already available from the data routinely collected in health IT systems [123].

Patient evaluations of the quality of care they receive in primary care settings can be appropriate complements to other measures of quality [106]. Patient assessments may be particularly useful for evaluating the quality of access, the practitioner-patient relationship, continuity, and coordination [106]. While findings from patient assessments, commonly conducted through questionnaires, may inform quality improvement efforts, it is not clear that the information gleaned from patients assessments can be successfully translated into actual improvements in the quality of care [106]. One study found that patient-reported satisfaction with quality of care among the elderly was not a good predictor of the effectiveness of the care these patients received [124]. However, ratings of coordination of care did have a relationship with survival time among the higher utilizers [124]. 

Studies conducted in the international setting have assessed how emphasis on primary care quality may impact health outcomes, and whether specialists or PCPs provide better quality of care for chronic conditions. The results were mixed. Improvements in health outcomes for diabetes type 2 patients in Norway may reflect special emphasis on improving diabetes care in primary care practices [125]. Research in Taiwan has found that patients with a PCP or a usual source of care (USC) experience superior primary care quality, including better access, coordination, family centeredness, continuity, and cultural competence [126, 127]. In Britain, an evaluation comparing the quality of diabetes care provided in specialist diabetes clinics to that provided by primary care clinics found no long-term difference in the rates of improvement in HbA1c, cholesterol, and blood pressure over time [128]. In contrast, a Danish study found that patients suffering from asthma or rhinitis experience superior care quality from respiratory specialists as opposed to PCPs [129]. In a study in Canada looking at asthmatic patients participating in an intervention designed to improve access to primary care, intervention patients did initially have better access in comparison to those patients without the primary care intervention [130]. However, after 12 months, there was no difference between the two groups [130]. In Canada, a small-scale preventive primary care outreach program targeting the elderly included home care, collaborative planning between patients, families and physicians, and referral to appropriate social support and community resources; the program, however, had no significant positive findings and no relationship with the functional status and self-rated health of participants [93].

Medical, contextual (specific to the medical encounter), and policy evidence is needed to further research on quality in primary care [131]. Cross-country comparisons may elucidate how broader systems-level factors impact primary care quality. Future studies should be undertaken to examine primary care reforms; how financing mechanisms impact PCP cooperation and workforce issues; the relationship between balance of primary and specialty care; the patient's role; community-oriented care; equity in access and outcomes [132].

In the USA specifically, Friedberg et al. [133] categorize the focus of proposed policy interventions intended to strengthen primary care and improve healthcare quality into three categories: (1) supply of PCPs; (2) the set of functions and services provided by a usual source of care; (3) the orientation of the health system. Based on a review of the evidence, these authors suggest that policy prescriptions should focus on reorienting the health system in the USA to emphasize and reward primary care provision and support providers' capacity for practicing primary care through such interventions as health IT, the Chronic Care Model, and team-based approaches to delivering care [133]. Although experts disagree over whether the current supply of PCPs impacts the overall quality of healthcare, some policy prescriptions for emphasizing primary care in the USA focus on increasing PCP supply by making the career more attractive through better wages [134] and through funding of Title VII Section 747, which supports workforce development [69, 70]. Grumbach and Mold also propose circulating a primary care version of an agricultural extension agent to disseminate the latest knowledge and clinical guidelines, improve the quality of care provided by PCPs, and strengthen the country's primary care infrastructure [135]. In addition, increased collaboration between primary care and public health may improve quality and health outcomes by refocusing family medicine on population or community health rather than just individuals [136]. In addition to these proposals, some point out that more policy actions should be devoted to distributing primary care resources more fairly and evenly [137]. Low-income, deprived communities are burdened by higher morbidity and mortality rates and, therefore, should receive more health services [137].

### 2.6. Primary Care and Cost

One consequence of having many specialists is the possibility that specialist care has contributed to increasing the volume of intensive, expensive, and invasive medical services and therefore the costs of healthcare [138–144]. Higher surgeon supply has been found to increase the demand for initial contacts with surgeons [145]. Many now frequently performed operations, such as coronary artery bypass, hip replacement, carotid endarterectomy, arthroscopy, laparoscopy, and heart and liver transplantation, were little known and hardly ever performed 50 years ago. Today, they are both fairly common and expensive.

Technological developments also drive up healthcare costs [146]. Systematic comparison across industrialized countries shows that the USA has higher rates of coronary surgery, diagnostic imaging, neurosurgery, treatment for end-stage renal disease, and cancer chemotherapy than any other country [147, 148]. As the disease prevalence for these conditions is still relatively low, an excess of specialists in these areas may lead to the performance of unnecessary procedures. The Congressional Subcommittee on Oversight and Investigations estimated that nationwide there were 2.4 million unnecessary operations performed annually, resulting in a cost of $3.9 billion and 11,900 deaths [149, 150]. Overall, primary care services are less costly than specialty services because they are less technology-intensive.

An economic analysis was conducted in the UK to assess the cost-effectiveness of Quality and Outcomes Framework (QOF) payments, which is an attempt to improve the quality of primary care in the UK through the use of financial rewards [151]. The study used 2004/2005 data on the QOF performance of all English primary care practices. Cost-effectiveness evidence was collected for a subset of nine QOF indicators with direct therapeutic impact. The authors found that the proportional changes required to make QOF payments cost-effective varied widely between the indicators. It showed that QOF incentive payments are likely to be a cost-effective use of resources for a high proportion of primary care practices, and incentive payments are likely to be a good value for the NHS.

Health policy experts suggest that systems, models, and providers oriented toward primary care may achieve lower healthcare costs, a top priority in the USA [52, 133]. The bulk of the evidence suggests that PCPs order fewer diagnostic tests and procedures than specialists, leading to lower costs [133]. In addition, having a usual source of care (defined as a primary care function, not explicitly as a PCP) is correlated with lower use of healthcare resources and lower rates of nonurgent emergency department visits, thus also decreasing costs [152]. On a systems level, regions of the USA with a higher PCP to specialist ratio experience not only better health outcomes but also lower costs [152]. Comparative analyses have found that other countries with health systems oriented toward primary care, on average, also have lower costs and better population health outcomes [152].

In addition to these more macro-level demonstrations of relationships between the primary care system and healthcare spending, cost-effectiveness studies and other forms of cost analyses can inform efforts to strengthen and improve primary care delivery [153]. One area of research explores what settings produce the best value or the highest quality care for the lowest costs. The community health center (CHC) program delivers primary care for vulnerable populations in areas identified by the Department of Health and Human Services' Health Resource and Services Administration as medically underserved [67]. A review of the literature on CHCs indicates that these centers provide quality primary care at low cost to especially disadvantaged populations [154]. However, the authors note that few studies have used formal cost-effectiveness methods to compare the value of CHCs to the value achieved in other primary care settings [154]. One recent study using Medicaid claims data to compare CHCs to other primary care settings found that while hospital outpatient departments and CHCs have similar costs, private physician practices actually have somewhat lower costs [155]. 

Some suggest that certain models of care may also lower costs. Staub, for instance, has argued that larger primary care group practices can apply management and technology innovations more fluidly than smaller practices, lowering the costs associated with these kinds of changes [156]. Others have looked to the patient-centered medical home (PCMH) as one model that shows promise in reducing healthcare spending. While the literature to date generally supports an association between the improved access and coordination of the PCMH model and reduced hospitalizations and ED visits, other predicted effects, such as decreased use of unnecessary tests, procedures, and referrals, have not yet been demonstrated [157]. One example of an integrated program, the Geisinger Health System's Proven Health Navigator (PHN), has led to cost savings of 4.3% to 7.1% [158]. The program's success in reducing costs may be an inspiration to other integrated delivery systems or primary care practices seeking to adopt the PCMH model [158]. Friedman et al. [152] also observe that health professionals who are not PCPs can perform primary care functions, an important consideration for the team-oriented PCMH model. Research suggests that care by nonphysicians, like physician assistants (PAs), for example, may be less costly than care by physicians [159].

Access to care may also impact costs. One study assessed a low-cost primary care physician access program's impact on ED use, noting that while the increased access may have prompted patients to change where they sought care for nonurgent purposes, the study could not demonstrate statistically significant cost savings [109]. This study and the evaluation of the Geisinger PCMH suggest that primary care interventions may require long periods of time to demonstrate financial savings [109, 158]. An eight-year study assessing the impact of a primary care case management (PCCM) program in Iowa's Medicaid program did demonstrate reductions in costs due to shifting expenses from the hospital to outpatient setting [160]. These savings increased over time, reinforcing what other studies suggest: these desired significant cost savings may take time to achieve [160]. Chernew et al. [161] explored how PCP/specialist supply influences cost, and the findings indicated that increasing the PCP supply may accrue a short-term advantage but does not address long-term problems. Although the proportion of the workforce comprised of PCPs in contrast to specialists likely affects healthcare expenditures, balancing the workforce in favor of PCPs may do little to curb the rate of growth in healthcare spending and thus will not reduce overall costs [161]. 

PCCM programs, like the one implemented for the Iowa Medicaid program, use the gatekeeper approach, in which patients select a PCP and are then required to obtain referrals through this PCP to see specialists, reducing unnecessary specialist appointments and procedures and therefore reducing healthcare costs. The PCP also may coordinate care for a panel of patients in a cost-effective manner [160]. 

Another intervention that has been touted as a potential way to reduce the costs of medical care in the USA is health IT. Research indicates that health IT systems may yield financial gains for PCPs by reducing drug expenditures, reducing utilization of expensive tests in favor of other equally useful diagnostic methods, and decreasing billing mistakes [162]. One study of the implementation of an electronic medical record (EMR) system in primary care clinics found cost savings that increased over time [162]. 

Other lines of research explore the cost-effectiveness of particular interventions conducted in the primary care setting [42, 98, 100, 163, 164]. For example, studies have explored the cost-effectiveness of different techniques designed to increase cancer screenings [42, 163], diabetes self-management programs [164], mental health interventions [98, 100], smoking cessation treatment [165], and lifestyle counseling and interventions [166], all within the primary care setting. This type of research seeks to inform quality improvement efforts in primary care with cost-effectiveness information. Ideally, PCPs could use this information to provide both higher quality and more efficient care.

### 2.7. Primary Care and Equity

Better primary care is also associated with more equitable distribution of health within a population [89, 90, 167, 168]. The annual National Healthcare Disparities Report in the USA [169] stated that equitable primary care eliminates disparities “related to preventive services and management of common chronic diseases typically delivered in primary care settings” [170]. Primary care providers deliver a disproportionate share of ambulatory care to disadvantaged populations. Improved access to primary care was associated with reduced mortality rates, better health outcomes, and lower costs [6, 171–175]. A higher proportion of PCPs in a given area has also been shown to lead to lower spending on healthcare [161]. Additionally, an increase of one primary care physician per a population of 10,000 is associated with a reduction of 1.44 deaths, a 2.5% reduction in infant mortality, and a 3.2% reduction of low birthweight on average in the population [167, 168, 176–179]. Such associations hold even in the presence of income inequality and other health determinants [172–175]. Adults who have PCPs as their regular source of care experience lower mortality and incur reduced healthcare costs [6]. 

Research has also shown that primary care may play an important role in mitigating the adverse health effects of income inequality [180–183]. Specifically, research has demonstrated associations between income inequality and self-rated health and primary care and self-rated health [181]. Therefore, the pathway through which income inequality impacts health may be partly attenuated by primary care [182]. Access to quality primary care may have the largest impact on health in areas with the highest levels of income inequality [182]. However, socioeconomic status may also reduce to some extent the impact of primary care on health [182]. The relationship between race, income inequality, primary care availability, and health is complicated; in stratified analyses of the impacts of primary care and income inequality on mortality, Shi and Starfield [183] found while independent associations between primary care and mortality and income inequality and mortality persisted after controlling for other socioeconomic variables among white Americans, the relationship between primary care physician supply and mortality lost its statistical significance with the inclusion of other socioeconomic factors in the model. 

Primary care availability may also be more strongly correlated with health outcomes in areas with greater levels of income inequality, suggesting that expanding primary care availability in these areas may have a substantial impact on population health [180]. However, only certain specialties under the umbrella of primary care may have this impact. For example, family medicine has been found to have the strongest inverse relationship with mortality [172–175]. These findings have been consistent in examinations of mortality at the state level [172–175], at the county level [167, 168], in comparisons of urban and nonurban areas [167, 168], and in stratifications by race [167, 168].

In the USA, racial and ethnic minorities face greater difficulty accessing regular primary care than white Americans and use hospitals more often than private clinics as usual sources of care [184]. Challenges included long wait times and difficulty obtaining timely appointments [184]. Addressing these barriers and ensuring more equitable access to high quality primary care may translate into reduced disparities in self-rated health status [176–178]. 

Access to primary care may have the greatest impact on health status for racial and ethnic minorities living in poverty [176–178]. However, research exploring why racial and ethnic minorities in the USA receive fewer preventive services determined that while frequency of visits to primary care physicians likely explains a small portion of the disparity, factors related to poverty are significantly more important [185]. While some research has suggested that physician-patient racial concordance may positively affect the quality of care racial/ethnic minority patients receive, other research has not borne out these conclusions [186].

A US study looking at the Latino population aimed to identify subgroup variations in having a patient-centered medical home, the PCMH's impact on disparities, and factors associated with Latinos having a PCMH in the USA [187]. The 2005 Medical Expenditure Panel Survey (MEPS) Household Component that sampled 24,000 adults, including 6,200 Latinos, was used in this analysis. Self-reports of preventive care and patient experiences were also examined. The results showed that white (57.1%) and Puerto Rican (59.3%) adults were most likely to have a PCMH, while Mexican/Mexican Americans (35.4%) and Central and South Americans (34.2%) were least likely. Much of this disparity was caused by lack of access to a regular provider. Respondents with a PCMH had higher rates of preventive care and positive patient experiences. Disparities in care were eliminated or reduced for Latinos with PCMHs. The regression models showed that private insurance, which is less common among Latinos than whites, was an important predictor of having a PCMH. These findings indicate that eliminating healthcare disparities will require assuring access to a PCMH and that addressing differences in healthcare coverage that contribute to lower rates of Latino access to the PCMH will also reduce disparities.

As seen in the previous study, insurance status is associated with access to primary care and the quality of that care [30]. The uninsured have greater difficulty accessing good primary care than the insured; among the insured, those with private insurance have better access to quality primary care than the publicly insured [30]. Those with health maintenance organization (HMO) plans have more comprehensive care but poorer measures of longitudinal and coordinated care than those in fee for service (FFS) plans [30].

Children also experience racial and ethnic disparities in access to and quality of primary care [188]. Stevens and Shi propose the following research agenda to explore health disparities in children further: (1) conduct research using more racial and ethnic granularity rather than categorizing groups superficially; (2) explore the role of language in contributing to health disparities; (3) consider cultural influences; (4) examine how health systems-level policies and factors contribute to disparities [188]. Recent attention has focused on how models, such as the patient-centered medical home, may improve quality of care for children. Yet research on the association between race and ethnicity and having a medical home has determined that minority children have lower odds of having healthcare experiences that contain features of the medical home, such as having a usual provider, a provider who spends sufficient time with him or her, and a provider who communicates well [189].

One unique population on which there is little literature is the migrant worker population. Part of the reason for this gap in research is the difficulty in determining how many migrant workers are living currently in the USA [190]. Although the federally qualified health center (FQHC) program currently provides primary care to an estimated 20% of agricultural workers, most migrant workers face significant structural barriers to accessing adequate primary healthcare [190]. 

The Patient Protection and Affordable Care Act may impact disparities in access to and quality of primary care by expanding insurance coverage and funding for FQHCs and the National Health Service Corps, which repays loans to physicians and health professionals practicing in shortage areas [191]. In addition, it includes innovations like the community-based collaborative care networks, which support low-income populations in accessing medical homes [191].

### 2.8. Primary Care and Health Centers

Creation of community health centers (CHCs)—formerly called neighborhood health centers—was authorized during the 1960s under the Johnson administration's War on Poverty program, seeking to address healthcare needs in medically underserved regions of the USA. The federal government determines the medically underserved designation to indicate a shortage of primary care providers and delivery settings, as well as poor health indicators for the population. Such areas are often characterized by economic, geographic, or cultural barriers that limit access to primary care for a large segment of the population. CHCs are required by law to locate in medically underserved areas and provide services to anyone seeking care, regardless of insurance status or ability to pay. Hence, CHCs are a primary care safety net for the nation's poor and uninsured in both inner city and rural areas.

CHCs operate under the Bureau of Primary Health Care (BPHC), Health Resources and Services Administration (HRSA), US Department of Health and Human Services (DHHS). Under Section 330 of the Public Health Service Act, CHCs receive some federal funds to provide primary care and services that improve access for disadvantaged populations, such as low-income groups, racial and ethnic minorities, public housing residents, the homeless [192], and migrant workers [190]. CHCs are private, nonprofit organizations that nonetheless depend heavily on funding through the Medicaid program and federal grants. Private-pay patients are charged on sliding-fee scales, determined by the patients' income.

CHCs tailor their services to family-oriented primary and preventive healthcare and dental services [193]. These centers have developed considerable expertise managing the healthcare needs of underserved populations. Many have developed systems of care that include outreach programs, case management, transportation, translation services, alcohol and drug abuse screening and treatment, mental health services, health education, and social services.

CHCs are governed by an executive director or administrator, have a medical director, and are staffed by multidisciplinary teams of clinicians. The typical CHC employs 6 PCPs, 8 nurses, and 3 NPPs. Some clinics also have dentists, mental health practitioners, and pharmacists on site. Other members of these teams may include case managers and education specialists [193].

In 2009, CHCs served 18.8 million individual patients in 74 million patient visits. The majority of groups that utilize CHCs are vulnerable populations—92% of patients were below the 200% federal poverty level and 38% were uninsured. Among special populations, more than 1 million homeless individuals, 865,000 migrant/seasonal farmworkers, and 165,000 residents from public housing were seen through the program [194]. 

Expanding the services of CHCs was a central element of President Bush's plan for expanding healthcare access to the uninsured and underserved. In 2002, the Bush Administration proposed a $1.5 billion budget to continue a long-term strategy that would fund 1,200 new and expanded CHC sites over 5 years and serve an additional 6.1 million patients [193]. The Obama Administration continued this growth through the American Recovery and Reinvestment Act of 2009, which allocated $2 billion to CHCs to expand their patient populations, create new jobs, and meet the increasing demand for primary care services [7].

Components of the Patient Protection and Affordable Care Act of 2010 that affect CHCs involve payment protections and initiatives to develop teaching health centers. The law ensures that CHCs are not underpaid for the services they provide and adds preventive services to the Medicare payment system, while eliminating Medicare payment caps. Additionally, the law allows for a Title VII grant program to develop residency programs in CHCs to teach the next generation of primary care providers [195]. This may be especially important because, although the ACA has increased CHC funding, health centers still face challenges in recruiting employees and maintaining financial viability. Rural centers, in particular, face personnel recruitment barriers [196, 197]. Research suggests that physicians who have exposure to medically underserved communities such as inner cities or rural areas of the country may be more inclined to practice in health centers located in these environments, making ACA efforts to offer residency training in these environments particularly prudent [198]. In addition to difficulties recruiting medical staff, CHCs have also faced fiscal challenges through the years. In the last several decades, as state Medicaid programs have increasingly relied upon managed care organizations to reduce costs, CHCs have engaged with managed care in order to maintain the important revenue obtained through Medicaid reimbursement [199]. However, research has found that CHC involvement with managed care also is associated with financial instability and serving fewer homeless and uninsured patients [199]. Therefore, federal funding of these centers remains imperative. 

Because the majority of the population served by CHCs belongs to vulnerable groups (i.e., low income, minorities, homeless), studies show potential for CHCs to help bridge the disparities faced by these populations [200–203]. A large body of research suggests that CHCs increase access to primary care for these populations [192, 193, 204, 205], reduce hospitalizations [192], and provide high quality care for these particularly challenging populations when compared to other providers and settings [172–175, 196, 197, 206]. In addition, CHCs may reduce disparities by race/ethnicity, income, or insurance status [31, 32, 176–178, 205, 207, 208]. Studies also suggest that the quality of care these populations receive at CHCs is the same as the quality provided by other primary care providers and that health center patients may incur lower inpatient costs [155, 209].

However, some disparities persist among health center patients. For example, the uninsured report poorer primary care experiences than Medicaid enrollees utilizing CHCs [205]. Generally, however, CHCs are an important avenue for accessing primary care for Medicaid and uninsured patients [193].

### 2.9. Primary Care and Healthcare Reform

A number of industrialized countries have embarked on healthcare reforms aimed, at least in part, at strengthening their primary care delivery systems. For example, a primary care reform in Quebec, Canada, was studied to see if/how patients' perceptions of the quality of care changed [210]. The study used a before-and-after comparison of the perceptions of patients to evaluate how primary care reform affected patients' experiences in primary care. A random sample of 1,046 participants from five family medicine groups (FMGs) in two regions of Quebec completed both the baseline and follow-up questionnaires. The authors found that perceptions of relational and informational continuity increased significantly, whereas organizational and first-contact accessibility and service responsiveness did not change significantly. Perception of physician-nurse coordination remained unchanged, but perception of primary care physician-specialist coordination decreased significantly. The proportion of participants reporting visits with nurses and reporting use of FMGs' emergency services increased significantly from baseline to followup. The findings showed that the reorganization of primary care services resulted in considerable changes in care practices, leading to improvements in patients' continuity of care but not to improvements in accessibility of care.

Another recent study assessed changes in patient experiences of primary care during health service reforms in England between 2003 and 2007 [211]. The researchers conducted a cross-sectional study of family practices in which questionnaires were sent to serial samples of patients in 42 representative general practices in England. Up to 12 patients with a confirmed diagnosis of each chronic illness (coronary heart disease, diabetes, or asthma) were randomly sampled in each practice. In addition, a random sample of 200 adult patients (excluding patients who reported any long-term condition) in each practice were also mailed a questionnaire. The results show that were no significant changes in quality of care reported by either group of patients between 2003 and 2007 regarding communication, nursing care, coordination, and overall satisfaction. Some aspects of access improved significantly for patients with chronic disease, but not for the patients without a long-term condition. The findings indicate that there were modest improvements in access to care for patients with chronic illness, but overall, patients now find it somewhat harder to obtain care, affecting care continuity. This outcome may be related to incorrect incentives to provide rapid appointments or to the increased number of specialized clinics in primary care. This research indicates that the possibility of unintended effects needs to be considered when introducing pay for performance schemes.

The impact of China's New Rural Cooperative Medical Scheme (NCMS) and its implications for rural primary healthcare were evaluated in a study that performed a difference-in-difference analysis to determine whether China's NCMS has corrected distortions in rural primary care and whether the policy has affected the operation and use of village health clinics [212]. A total of 160 village primary care clinics and 8,339 individuals within 25 rural counties across five Chinese provinces were involved in this study. The study sought to evaluate the effect of NCMS by using individual level and village clinic level data collected in 2004 (shortly after the introduction of the scheme in selected regions) and in 2007 (after the dramatic expansion of the scheme across most rural areas). For individuals, NCMS is not clearly related to the use of medical care, but it may have redirected patients away from specialized facilities to village clinics. On the clinic level, NCMS has increased clinics' weekly patient flow and gross income, but not annual net revenue. Increases in patient flow and gross, but not net, clinic income may reflect desirable reductions in the provision of specialized, high profit services and rates of drug sales.

## 3. Conclusion

Primary care is imperative for building a strong healthcare system that ensures positive health outcomes, effectiveness and efficiency, and health equity. It is the first contact in a healthcare system for individuals and is characterized by longitudinality, comprehensiveness, and coordination. It provides individual and family-focused and community-oriented care for preventing, curing or alleviating common illnesses and disabilities, and promoting health.

Many countries in the world have embraced primary care, using a variety of structures and models. Lessons from these countries could serve as case studies for the US healthcare system, which currently faces an imbalance between specialty and primary care as well as a significant shortage and inequitable distribution in the primary care workforce. Different types of indicators and tools have been developed to measure the function of primary care, the performance of providers and facilities, quality of care, and so forth, but the need for more indicators and more data continues. Patient-centered measurements are gradually replacing disease specific measurements to yield more accurate assessment of primary care.

In both developed and developing countries, primary care has been demonstrated to be associated with enhanced access to healthcare services, better health outcomes, and a decrease in hospitalization and use of emergency department visits. Primary care can also help counteract the negative impact of poor economic conditions on health. Therefore, research suggests the need to increase the supply of primary care physicians in the USA. Further research is also needed to evaluate what models of primary care can produce the best health outcomes.

There are many factors determining quality of care, such as ease of access (including availability of after-hours care, length of office wait time, travel time to an appointment, and flexibility in selecting a PCP), clinical quality, interpersonal aspects, continuity, structure through which primary care is delivered, and insurance coverage. Although studies in international settings have compared quality of care in primary care and specialty care settings, the results were mixed, and further research is needed to elucidate how system-level factors, and certain policies may influence quality in the USA.

In addition, research has indicated that countries and regions more oriented to primary care have lower healthcare costs but better health outcomes, although further studies using formal cost-effectiveness methods need to be conducted. Cost-effectiveness of primary care has been tentatively established through a few interventions conducted in primary care settings, and adoption of health information systems in primary care settings may further yield financial gains.

Furthermore, better primary care is correlated with more equitable distribution of health within a population and can mitigate the adverse effects of income inequality, which is especially important in the USA where racial and ethnic minorities face greater difficulties accessing regular primary care. This in turn emphasizes the significant role of CHCs in the USA in providing primary care services to vulnerable groups and reducing disparities. CHCs in the USA are primary care facilities that provide family-oriented services to meet the healthcare needs of medically underserved populations. However, difficulties in recruiting primary care providers and maintaining financial viability are major challenges to the sustainability of CHCs, which subsequently influences primary care services available to and health outcomes for these underserved populations. Additionally, research on health disparities in children and migrant workers is still lacking and needs further attention.

Lastly, healthcare reforms aimed at strengthening the primary care system have been implemented in a number of countries, both developed and developing, and have generally proven to improve the healthcare system as a whole. The Patient Protection and Affordable Care Act (ACA) also emphasizes primary care in the USA. Future assessments focusing on the impact of the ACA on primary care, health outcomes, healthcare costs, and health disparities should be conducted to serve as an empirical basis for policy making in the future.
